# Cetirizine per os: exposure and antihistamine effect in the dog

**DOI:** 10.1186/s13028-018-0431-3

**Published:** 2018-11-26

**Authors:** Carl Ekstrand, Carina Ingvast-Larsson, Ulf Bondesson, Mikael Hedeland, Lena Olsén

**Affiliations:** 1 Department of Biomedicine and Veterinary Public Health, Division of Pharmacology and Toxicology, Swedish University of Agricultural Sciences, P.O. Box 7028, 750 07 Uppsala, Sweden; 2 Department of Chemistry, Environment and Feed Hygiene, National Veterinary Institute, Uppsala, Sweden; 3 Department of Medicinal Chemistry, Division of Analytical Pharmaceutical Chemistry, Uppsala University, Uppsala, Sweden; 4 Department of Clinical Sciences, Division of Veterinary Nursing, Swedish University of Agricultural Sciences, Uppsala, Sweden

**Keywords:** Anti-inflammatory, Efficacy, Pharmacodynamics, Pharmacokinetics, Potency

## Abstract

**Background:**

Cetirizine is an antihistamine used in dogs, but plasma concentrations in relation to effect after oral administration are not well studied. This study investigated cetirizine exposure and the plasma cetirizine concentration-antihistamine response relation in the dog following oral administration of cetirizine.

**Results:**

Eight Beagle dogs were included in a cross-over study consisting of two treatments. In treatment one, cetirizine 2–4 mg/kg was administered per os once daily for 3 days. The other treatment served as a control. Wheal diameter induced by intra-dermal histamine injections served as response-biomarker. Cetirizine plasma concentration was quantified by UHPLC-MS/MS. Median (range) cetirizine plasma terminal half-life was 10 h (7.9–16.5). Cetirizine significantly inhibited wheal formation compared with the premedication baseline. Maximum inhibition of wheal formation after treatment with cetirizine per os was 100% compared with premedication wheal diameter. The median (range) *IC*_50_-value for reduction in wheal area was 0.33 µg/mL (0.07–0.45). The median (range) value for the sigmoidicity factor was 1.8 (0.8–3.5). A behavioral study was also conducted and revealed no adverse effects, such as sedation.

**Conclusion:**

The results indicate that a once-daily dosing regimen of 2–4 mg/kg cetirizine per os clearly provides a sufficient antihistamine effect. Based on this experimental protocol, cetirizine may be an option to treat histamine-mediated inflammation in the dog based on this experimental protocol but additional clinical studies are required.

## Background

Inverse histamine 1 (H_1_) receptor-agonists, usually referred to as H_1_-receptor antagonists or antihistamines, are frequently used to prevent allergic reactions in humans [1–3]. Antihistamines can be divided into first generation antihistamines (e.g. hydroxyzine) and second generation antihistamines (e.g. cetirizine). First generation antihistamines are known to produce sedation due to antihistamine effects on the central nervous system [4]. In contrast, sedation is not as common among the effects of second-generation antihistamines due to their higher affinity to the efflux protein P-glycoprotein (Pgp) in the blood–brain barrier [5–7]. There are currently few antihistamines labeled for use in dogs in Europe. However, antihistamines have been prescribed off-label with little available scientific data on the pharmacokinetics (PK) and pharmacodynamics (PD) available. Both PK and PD might vary between species, so efficacy in humans is not a guarantee of efficacy in dogs. For instance, the antihistamines diphenhydramine and clemastine have low bioavailability and clemastine was demonstrated to have low antihistamine effect due to low systemic exposure after oral administration in dogs [8, 9]. Compared with clemastine, the antihistamine response to the second generation antihistamine cetirizine (1 mg/kg once daily) in the dog is more efficacious, around 30–80% with a marked variations between animals and between studies [10, 11]. This can be compared with the antihistamine response after oral administration of 2 mg/kg hydroxyzine, which is around 90% and shows lower inter-individual variability [12]. Hydroxyzine is metabolized to cetirizine, which is considered the active substance to the antihistamine response after hydroxyzine administration. In the studies by Temizel et al. [10] and De Vos et al. [11] plasma concentrations of cetirizine were not reported, so the most probable explanation for the conflicting results for hydroxyzine and cetirizine are variations in systemic exposure of cetirizine due to the low dose and perhaps low bioavailability. In addition, there are no data available that describe the plasma concentration–time course of cetirizine and linking the cetirizine exposure to the response using pharmacokinetic/pharmacodynamic (PK/PD) models. The aims of this study were thus to investigate exposure of cetirizine administered per os, to link the exposure to the antihistamine effect and to indicate apparent adverse effects.

## Methods

This study was approved by the Animal Ethics Committee, Uppsala, Sweden (C100/14).

Eight female Beagle dogs, 4–7 years old and weighing 10.0–13.5 kg, were given racemic cetirizine dihydrochloride (Cetirizin Sandoz 10 mg, Sandoz AS, Copenhagen, Denmark) as tablets in meat-balls or a control treatment (meat-balls) orally in a two-treatment cross-over design. Cetirizine was administered at 0, 24 and 48 h. The first administered dose was 4 mg/kg body weight. The second and third administered doses were 4 mg/kg in four dogs and 2 mg/kg in four dogs. Blood was collected in heparinized tubes before cetirizine was administered at 0, 24, 48 h and at 50, 51, 52, 55, 57, 59, 72, 76, 81 and 96 h. Blood samples were centrifuged at 1500*g* for 10 min (+ 4 °C). The plasma was then stored at − 70 °C pending analysis.

### Recording of the pharmacodynamic response to cetirizine exposure

Wheal-formation induced by intradermal histamine injection (0.07 mL of 0.1 mg/mL solution) was used to evaluate the antihistamine response. Before the start of each treatment, the dogs were bilaterally shaved on the thorax with electric clippers. A total volume of 0.07 mL of histamine hydrochloride (Soluprick, 10 mg/mL, ALK-Abello A/S, Horsholm, Denmark) diluted in saline (Natriumklorid Fresenius Kabi 9 mg/mL, Fresenius Kabi AB, Uppsala, Sweden) to a final concentration of 0.1 mg/mL was injected using a 0.4 × 19 mm (27 gauge) needle before each blood sample. The diameter of the wheal induced by histamine was determined 20 min after histamine injection, as the mean of two perpendicular diameters measured using a digital Vernier caliper. Two injections were performed at each time-point and the largest reaction was used for further calculations. Sterile saline (0.07 mL) served as a negative control.

### Behavioral study

To detect any adverse effects such as sedation a behavioral study was conducted. The dogs were filmed when kept in their home environment at 1.5–2.5 and 3.0–4.5 h after drug administration on days one and two of the trial. The films were analyzed and behaviors were recorded according to two different protocols: The total time that each dog performed the behaviors drinking water, urinating, defecating, panting, yawning, barking, playing and licking (body, paws or lips) was calculated. Frequency of barking, lip licking and shaking the body was also noted.

For each dog, the behaviors sitting, lying, and active (including walking, jumping and standing) were also recorded during 4 min in every 20 min of films. Behaviors at 12 sample points at 20 s intervals within the 4 min period were recorded on score sheets.

### Analytical method

Determination of cetirizine was performed at the National Veterinary Institute (SVA) in Uppsala, Sweden. The sample pretreatment for plasma was as follows: To 100 µL of Li-heparin plasma (calibrators, QCs or study samples), 50 µL of the internal standard solution containing ^2^H_4_-cetirizine (0.11 µg/mL) were added. For protein precipitation, 100 µL of trichloroacetic acid (20%, w/v) were added, and the samples were mixed for 10 min followed by centrifugation for 5 min at 10,000*g*. The supernatants were transferred to vials and 10 µL of each sample were injected into an ultra-high-performance liquid chromatography-tandem mass spectrometry (UHPLC-MS/MS) system composed of an Acquity UPLC coupled to a Quattro Ultima Pt tandem quadrupole mass spectrometer with an electrospray interface operating in the positive mode (Waters Corporation, Milford, MA). The column was an Acquity UPLC BEH C18 (length 100 mm, I.D. 2.1 mm, particle size 1.7 µm) kept at 60 °C. The mobile phase consisted of (A) 10 mM ammonium formate in water and (B) 0.1% formic acid in acetonitrile. The elution was carried out as follows: initially at 20% B for 1.0 min, increase to 90% B during 1 min, constant at 90% B for 1.0 min, decrease to 20% B during 0.1 min, constant at 20% B for 1.9 min. The total run time was 5.0 min and the flow-rate was 400 µL/min. The analyte was quantified using a positive capillary voltage of 0.90 kV and a cone voltage of 35 V. The desolvation and source block temperatures was 300 °C and 120 °C, respectively, and desolvation gas flow was 950 L/h. The quantification was performed in the selected reaction monitoring (SRM) mode with the collision cell filled with argon gas at a pressure of 1.95 × 10^−3^ mBar. The mass transitions used in SRM were m/z 389 → 201 for cetirizine (collision energy 20 eV) and m/z 393 → 201 for [^2^H_4_]-cetirizine (collision energy 20 eV). The dwell time was 0.100 s. The reference standard cetirizine and the internal standard [^2^H_4_]-cetirizine were both obtained from Toronto Research Chemicals (North York, ON, Canada). The calibration curves were constructed using the chromatographic peak area ratio (analyte/internal standard) as a function of analyte concentration. The calibration functions were calculated by linear curve fit using a weighting factor of 1/x^2^. The calibration range was 0.3–10,000 ng/mL and the precision (relative standard deviation) was in the range of 0.6–7.3% and the accuracy was 98–114%.

### Data analyses

**Fig. 1:**
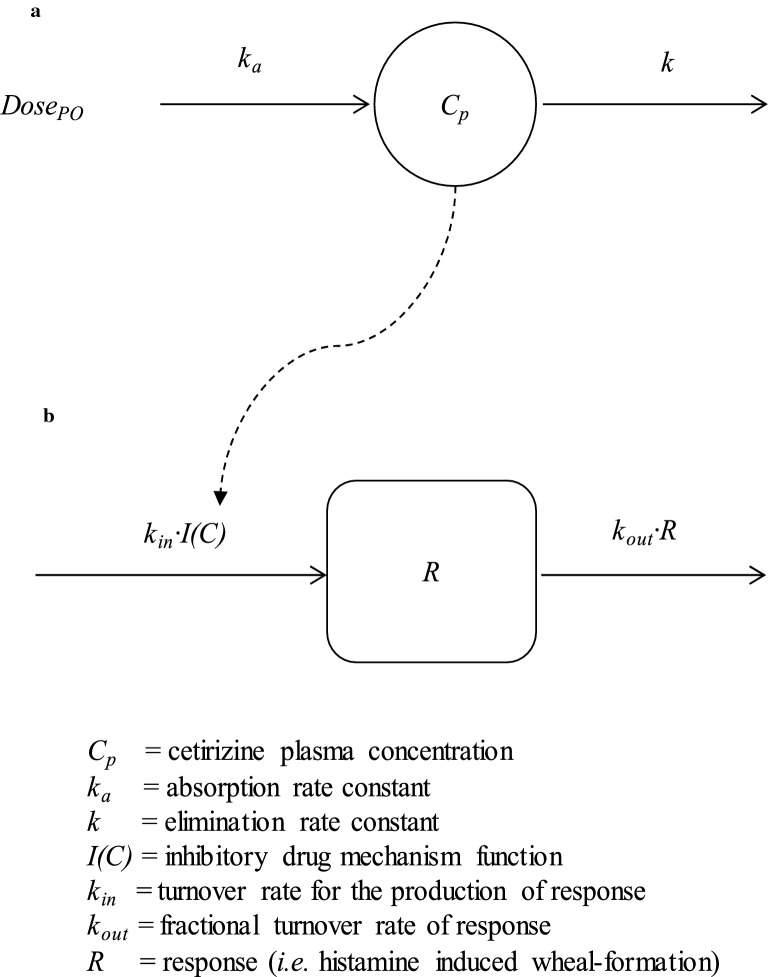
**a** The one-compartment model used to describe the plasma concentration–time course of cetirizine after oral administration (Eq. 1). **b** The pharmacodynamic model where the plasma exposure function served to ‘drive’ the drug mechanism function acting on histamine-induced wheal formation in Eq. 4

A one compartment model was fitted to experimental cetirizine data from each dog in order to predict and describe the cetirizine plasma concentration–time course. Individual concentration–time profiles were then used in an inhibitory function and ‘drove’ a turnover model in the PK/PD analyses (Fig. 1). The cetirizine plasma concentration–time course was described as:

1 $$C_{p} = \frac{{k_{a} \cdot F \cdot Dose_{po} }}{{V \cdot (k_{a} - k)}} \cdot \left[ {e^{{ - k \cdot (t - t_{lag} )}} - e^{{ - k_{a} \cdot (t - t_{lag} )}} } \right]$$

where *C*_*p*_ is the plasma concentration of cetirizine and Dose_po_ is the dose administered per os. The model parameters were *V/F*, which is the ratio between volume and the bioavailability, *t* and *t*_*lag*_ represent time and the lag time, respectively, and *k*_*a*_ and *k* are the absorption rate constant and the elimination rate constant, respectively. The terminal half-life (*t*_*1/*2z_) of cetirizine in plasma was calculated as

2 $$t_{1/2z} = \frac{\ln (2)}{k}$$

Cetirizine was assumed to directly inhibit histamine induced weal formation described as

3 $$I(C_{p} ) = 1 - \frac{{C_{p}^{n} }}{{IC_{50}^{n} + C_{p}^{n} }}$$

where *I(C)*, *IC*_50_ and *n* are the inhibitory drug mechanism function, the cetirizine plasma concentration at 50% reduction of the response and the sigmoidicity factor, respectively. The turnover of histamine-induced wheal formation with the inhibitory drug mechanism incorporated was described by

4 $$\frac{dR}{dt} = k_{in} \cdot \left[ {1 - \frac{{C_{p}^{n} }}{{IC_{50}^{n} + C_{p}^{n} }}} \right] - k_{out} \cdot R$$

where $\frac{dR}{dt}$ is the rate of change over time, *k*_*in*_ and *k*_*out*_ are the turnover rate for the production of response and the first-order fractional turnover rate for loss of response, respectively, and *R* is the response.

### Model evaluation

One- and two compartment models with and without lag-time were fitted to the data. The most appropriate model was chosen based on visual inspection of diagnostic plots, objective function values, Akaike Information Criterion (AIC) and Schwarz Criterion (SC).

### Statistics

The effect of treatment was subjected to statistical hypothesis testing by means of a one-sided Wilcoxon Rank Sum test for paired data observations. Statistical significance was considered when P < 0.004 (Šisák-corrected P-value for repeated measurements).

The behavioral data were subjected to statistical hypothesis testing by means of a two-sided Wilcoxon Rank Sum test for paired data observations. Statistical significance was considered when P < 0.05.

All statistical analyses were performed using the statistical software R version 3.4.0 (The R Foundation for Statistical Computing, Vienna, Austria).

## Results

### Cetirizine concentration–time course analyses

**Fig. 2:**
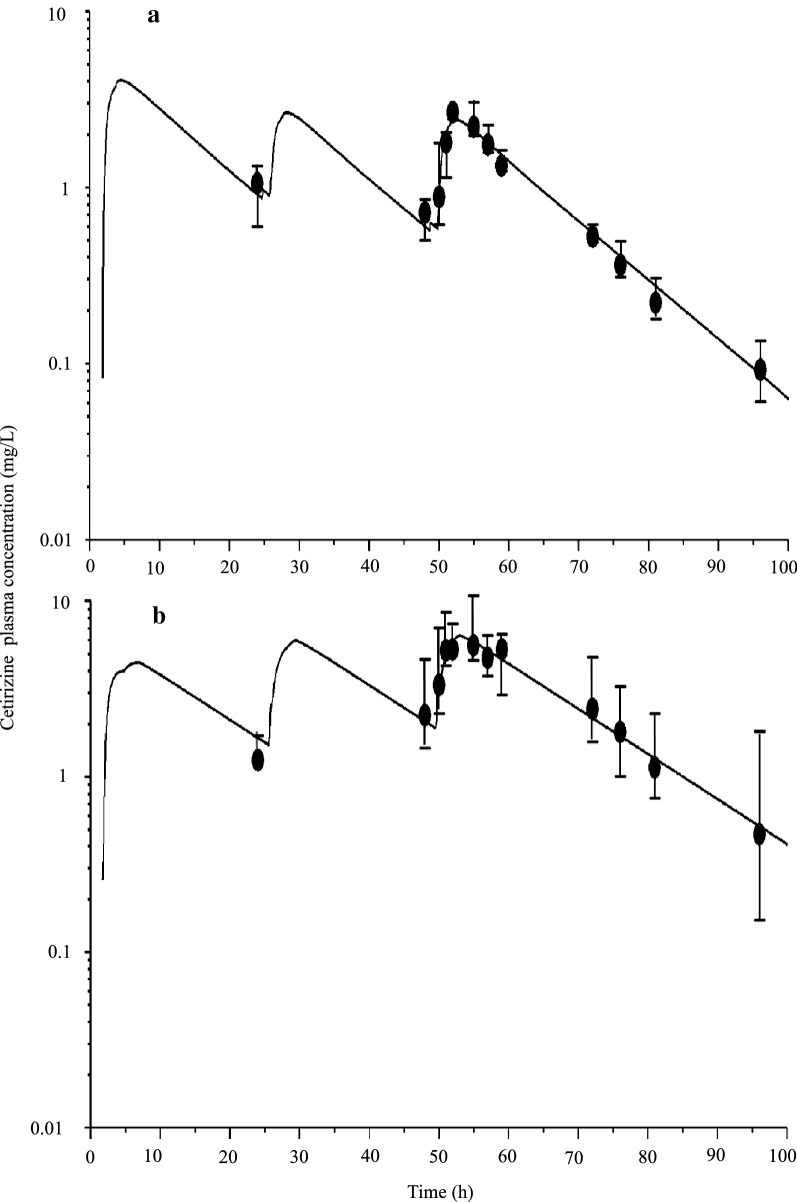
Median (range) observed (symbols) and median predicted (lines) plasma cetirizine concentrations in Beagle dogs over time after oral administration of cetirizine (4 mg/kg followed by 2 mg/kg once daily (n = 4) for an additional 2 days (upper plot, **a**) or 4 mg/kg once daily (n = 4) for 3 days (lower plot, **b**)

After treatment with 4 mg/kg cetirizine followed by 2 mg/kg cetirizine once daily, median (range) maximum observed plasma concentration was 2.7 µg/mL (2.5–2.8) and observed 4 h after the last administration of cetirizine (Fig. 2). Before cetirizine was administered at 24 h and 48 h, median (range) plasma concentrations were 1.1 µg/mL (0.6–1.3) and 0.7 µg/mL (0.5–0.8), respectively. After treatment with 4 mg/kg cetirizine once daily for 3 days, median (range) maximum observed plasma concentration was 5.6 µg/mL (4.6–10.8) and observed 7 h after the last administration of cetirizine. Before cetirizine administration at 24 h and 48 h, median (range) plasma concentration was 1.3 µg/mL (1.2–1.7) and 2.2 µg/mL (1.5–4.7), respectively. The median (range) model parameter estimates for $\frac{V}{F}$, *k*_*a*_, *k* and *t*_*lag*_ were 0.8 (0.6–0.9), 1.0 per h (0.4–4.0), 0.07 per h (0.04–0.09) and 1.8 h (0.7–2.8), respectively. The plasma half-life was 10 h (7.9–16.5).

### Wheal diameter-time course

**Fig. 3:**
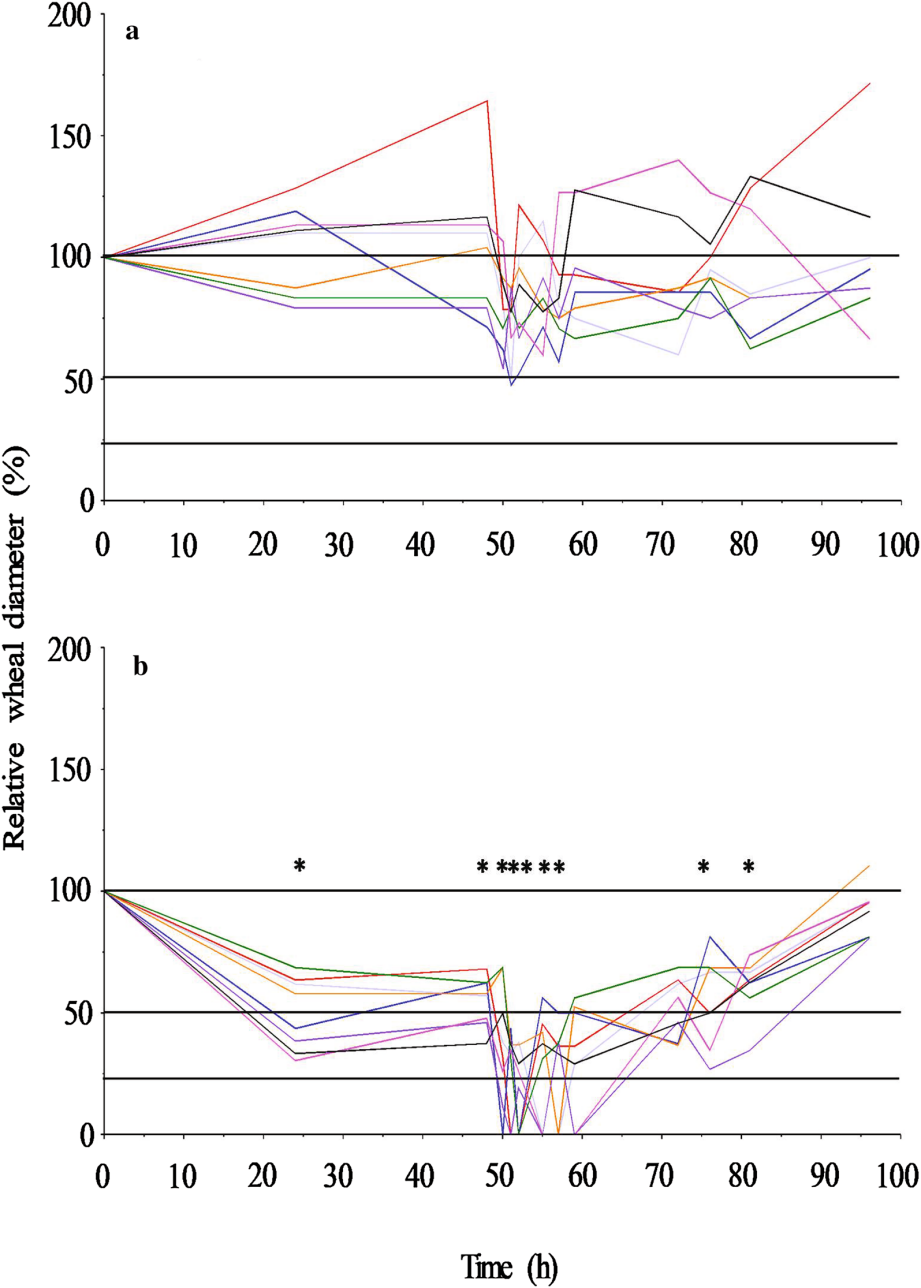
Histamine-induced wheal diameter over time relative to pre-medication values in eight dogs receiving either control treatment (upper plot, **a**) or treatment with cetirizine (lower plot, **b**) per os. * denotes statistically significant (P < 0.004, the Šisák-corrected P-value for repeated measurements) smaller diameter after treatment with cetirizine compared with the control treatment

Compared with the pre-medication wheal diameter, median (range) relative wheal diameters was 88% (48–171%) in the control treatment and 46% (0–111%) when the dogs were treated with cetirizine (Fig. 3).

**Fig. 4:**
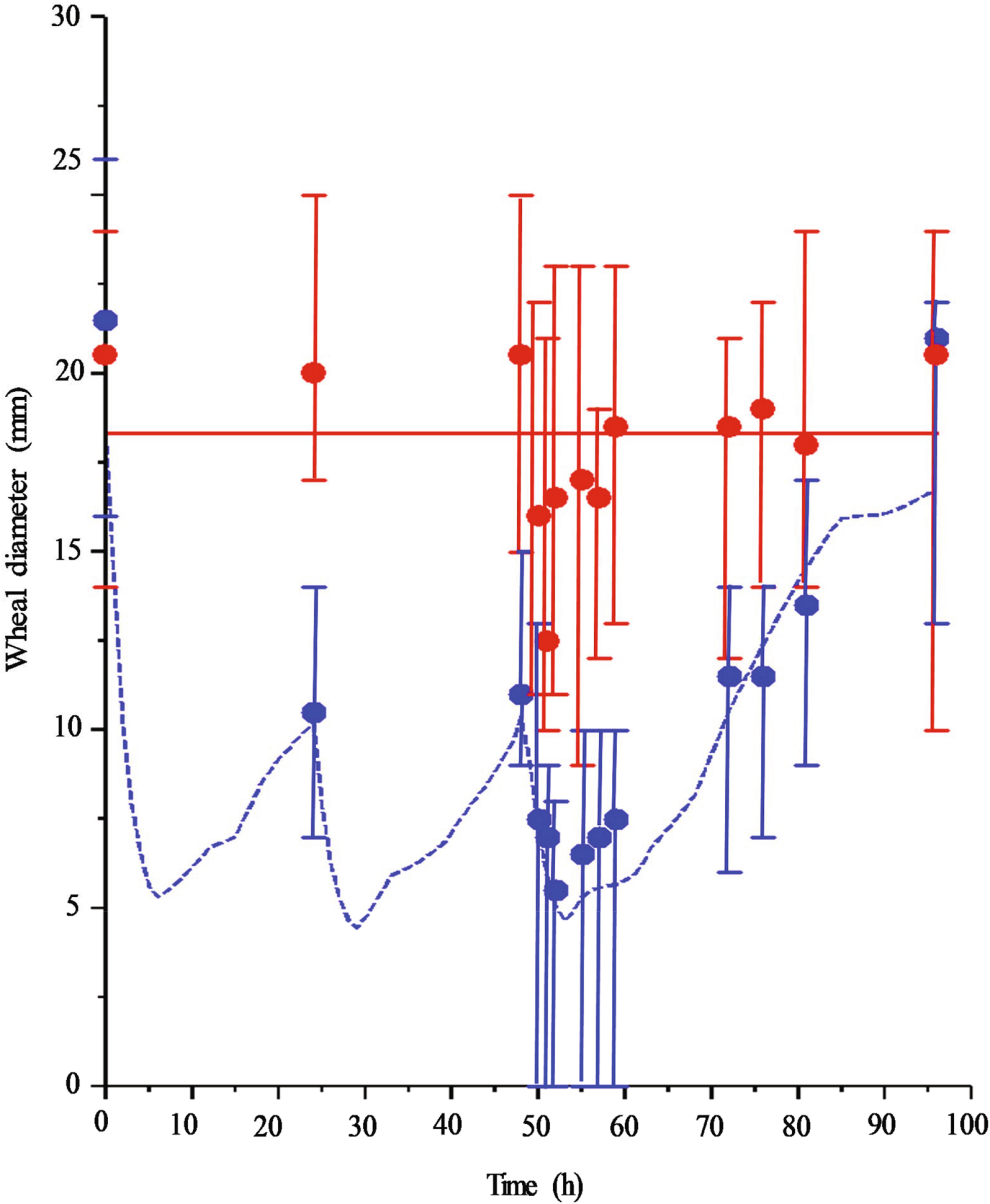
Median (range) observed (symbols) and median predicted (lines) histamine induced wheal-diameters over time after oral treatment with cetirizine (blue circles, blue dashed line) or control treatment (red circles, red solid line) in a cross-over study including eight dogs

There was no difference in wheal diameter between treatment with cetirizine and control treatment at time = 0 (P = 0.73). However, wheal diameter was significantly (P < 0.004, Šisák-corrected P-value) smaller after treatment with cetirizine from 24 h (the first observation after cetirizine administration) to 57 h and from 76 h to 81 h (the penultimate observation) compared with the control treatment. The P-value at 59 h and 71 h was P = 0.007 and P = 0.02, respectively. There was no significant difference between the two treatments at the final observation (96 h, P = 0.53). The variation in wheal diameter between animals was lower after treatment with cetirizine than after control treatment (Fig. 3). There was no difference in wheal response between the two cetirizine dosing protocols (4, 2, 2 mg/kg and 4, 4, 4 mg/kg). The PD model fitted to experimental wheal data accurately mimicked the wheal diameter-time course (Fig. 4). The median (range) *IC*_50_-value for the reduction of wheal area was 0.33 µg/mL (0.07–0.45). The median (range) value for the sigmoidicity factor (*n*) was 1.8 (0.8–3.5).

### Behavioral study

No differences in total time of different behaviors were noted. The frequency of lip licking was sufficiently high in six dogs and to be analyzed statistically, but was found not to be significantly different when the dogs were treated with cetirizine compared with the control-treatment. For the behaviors sitting, lying, and activity that were observed every 20 min, only four dogs could be included in the statistical analysis. Those dogs were visible on the video film for 70–100% of the observation period. One dog was moved to another compartment and three dogs were not visible on the video for more than 50% of the observed time. Those four dogs were excluded from the analyses. No significant differences could be established for any of these behaviors.

## Discussion

This study is the first to report plasma concentrations of cetirizine, the concentration–time profile of cetirizine and PD parameters for the antihistamine response after administration of cetirizine per os in the dog. The results indicate that systemic exposure to cetirizine after oral treatment may inhibit histamine-induced wheal formation by up to 100% (Fig. 3). The antihistamine response in this study is consistent with the reported antihistamine response to cetirizine after administration of 2 mg/kg hydroxyzine per os daily in dogs, while medication with 1 mg/kg cetirizine orally once daily results in a lower antihistamine response [10, 12]. These conflicting results may be explained by insufficient plasma exposure when lower doses of cetirizine than in this study are used. Due to the fairly long half-life (median 10 h) of cetirizine, 2 mg/kg daily dose is sufficient to maintain plasma concentration exceeding the median *IC*_50_ value (0.33 µg/mL) for 24 h. This quantitative information is similar to the median half-life of cetirizine (9.7 h) and the *IC*_50_-value (0.6 µg/mL), after administration of hydroxyzine, reported by Bizikova et al. [12].

Antihistamines are widely used in human medicine. When used in dogs with atopic dermatitis, some dog owners report satisfactory improvement of the clinical signs [13]. In clinical studies, the outcome of treatment with antihistamine is lower: 0–30% of patients are satisfactorily improved [14–17]. The very sparse available PK/PD data on antihistamines in the dog make interpretation of these data difficult and it is impossible to evaluate whether the dose produces a pharmacologically relevant exposure. From this perspective, further experimental PK/PD-studies on antihistamines, combined with drug concentration assessment in plasma at the time of observation in clinical studies, are warranted.

In clinical studies with high quality scientific evidence (i.e. randomized, blinded and placebo controlled), the results are conflicting [17, 18]. Hsiao et al. [18] were unable to demonstrate any difference in pruritus score between cetirizine and control treatments in a study population of 50 dogs whereas Eichenseer et al. [17] reported reduction of pruritus after medication with antihistamines (dimethindene or chlorpheniramine/hydroxyzine) in a cross-over study using a study population of 20 dogs. The limited number of dogs included in those studies lowers the statistical power and discrete improvements may have been missed. Despite this, the antihistamine combination chlorpheniramine/hydroxyzine in the latter study significantly decreased skin lesions, an effect not observed after treatment with dimethindene. After pretreatment for 24 h, neither 0.5 mg/kg cetirizine nor 2 mg/kg hydroxyzine was effective in preventing skin lesions in experimentally induced dermatitis [19]. A dose of 0.5 mg/kg cetirizine is unlikely to give plasma concentrations above the potency (*IC*_50_) value. Hydroxyzine at 2 mg/kg prevents wheal and flare reactions due to intradermal skin injections, mainly due to the effects of the active metabolite cetirizine [12]. The reduction in skin lesion score shown in the study by Eichenseer et al. [17] may be due to the higher total dose of antihistamines given by means of combining two different antihistamines. However, the effect of antihistamines on clinical signs of atopic dermatitis is likely to be limited. It is most likely that inflammatory mediators other than histamine are involved in canine atopic dermatitis and that the efficacy of antihistamines is greater when given as a prophylactic to prevent the effects of released histamine [20]. Cetirizine should therefore be administered before onset of clinical signs and preferentially in patients with milder symptoms [21]. When the disease is already established, the antihistaminic response to cetirizine is limited to being an adjunct to other therapies in the control of atopic dermatitis [20, 22].

No adverse effect was observed in our behavioral study. One of the most common unwanted responses to antihistamines in humans is dry mouth, which is produced by an antagonistic effect on muscarinic receptors [23, 24]. This was not observed in this study in dogs, in which both drinking and licking the lips might indicate dry mouth as these behaviors were not different between the treatment with cetirizine and control treatment. These results are reasonable in light of the fact that cetirizine has low affinity for muscarinic receptors and high affinity and high selectivity for H_1_-receptors [25]. Sedation could be another possible unwanted response. Clinical experience and experimental studies both indicate that hydroxyzine can produce light sedation in dogs [12]. However, cetirizine is a substrate for Pgp and produces less sedation than antihistamines, which are not and therefore enter the CNS to a larger extent [6]. In the behavioral study reported here, no significant differences were seen in behaviors thought to indicate the degree of sedation (sitting, lying, and active) during the different treatments. This is consistent with the low frequency of sedation reported in clinical studies on both humans and dogs [15, 18, 23].

## Conclusions

Cetirizine at 2 mg/kg daily is effective in preventing wheal formation induced by intradermal histamine injections without any obvious unwanted effect. The long half-life of cetirizine in plasma results in plasma cetirizine concentrations that are likely be maintained above the *IC*_50_-value for wheal-inhibition using a once-daily dosing regimen. Therefore, cetirizine has the potential to prevent the symptoms of allergic responses mediated by histamine. It is unlikely to be effective against established atopic dermatitis as sole therapy but may prove beneficial as an adjunct to other medication.
